# Target of Rapamycin Complex 1 (TORC1), Protein Kinase A (PKA) and Cytosolic pH Regulate a Transcriptional Circuit for Lipid Droplet Formation

**DOI:** 10.3390/ijms22169017

**Published:** 2021-08-20

**Authors:** Vitor Teixeira, Telma S. Martins, William A. Prinz, Vítor Costa

**Affiliations:** 1 Yeast Signalling Networks, i3S—Instituto de Investigação e Inovação em Saúde, Universidade do Porto, 4200-135 Porto, Portugal; telma.martins@ibmc.up.pt (T.S.M.); vcosta@ibmc.up.pt (V.C.); 2 Yeast Signalling Networks, IBMC—Instituto de Biologia Molecular e Celular, Universidade do Porto, 4200-135 Porto, Portugal; 3 ICBAS—Instituto de Ciências Biomédicas Abel Salazar, Universidade do Porto, 4050-313 Porto, Portugal; 4 Laboratory of Cell and Molecular Biology, National Institute of Diabetes and Digestive and Kidney Diseases, NIH, Bethesda, MD 20892, USA; williamp@intra.niddk.nih.gov

**Keywords:** lipid droplet, membrane biogenesis, nutrient, cell signaling, transcription

## Abstract

Lipid droplets (LDs) are ubiquitous organelles that fulfill essential roles in response to metabolic cues. The identification of several neutral lipid synthesizing and regulatory protein complexes have propelled significant advance on the mechanisms of LD biogenesis in the endoplasmic reticulum (ER). However, our understanding of signaling networks, especially transcriptional mechanisms, regulating membrane biogenesis is very limited. Here, we show that the nutrient-sensing Target of Rapamycin Complex 1 (TORC1) regulates LD formation at a transcriptional level, by targeting *DGA1* expression, in a Sit4-, Mks1-, and Sfp1-dependent manner. We show that cytosolic pH (pHc), co-regulated by the plasma membrane H+-ATPase Pma1 and the vacuolar ATPase (V-ATPase), acts as a second messenger, upstream of protein kinase A (PKA), to adjust the localization and activity of the major transcription factor repressor Opi1, which in turn controls the metabolic switch between phospholipid metabolism and lipid storage. Together, this work delineates hitherto unknown molecular mechanisms that couple nutrient availability and pHc to LD formation through a transcriptional circuit regulated by major signaling transduction pathways.

## 1. Introduction

Lipid droplets (LDs) are ubiquitous organelles that store and supply lipids for energy homeostasis and membrane synthesis. They are made of neutral lipids (NL), triacylglycerols (TAG), and sterol esters (SEs), surrounded by a single monolayer of phospholipids with various decorating proteins [1,2,3]. In yeast, the synthesis of neutral lipids takes place in the ER bilayer and LD surface [1,2,3]. Acyl coenzyme A (CoA):sterol O-acyltransferase (ASAT) Are1 and Are2 catalyze the esterification of ergosterol with fatty acids (FAs), whereas Dga1 and Lro1, with acyl-CoA:diacylglycerol acyltransferase (DGAT) activity, are responsible for TAG generation. Surprisingly, the signaling events regulating LD metabolism in response to nutrient and physiological cues remain largely unknown. Understanding those molecular mechanisms is critical for the development of novel strategies aimed at treating diseases associated with impaired LD dynamics, as aberrant LD metabolism is a hallmark of several disorders, including diabetes and neurodegeneration [4,5].

**Figure 1:**
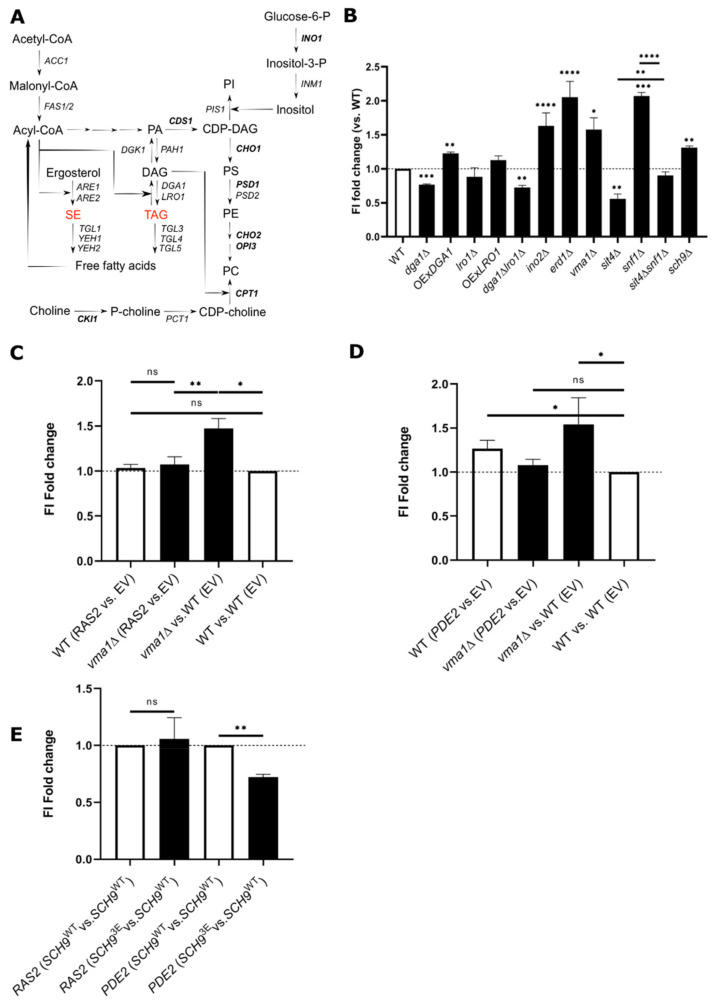
Mutants in nutrient sensing pathways have altered LD metabolism. (**A**) Overview of lipid metabolism in yeast. The set of genes regulated by major transcription factor Opi1 are highlighted in bold. (**B**) Inhibition of TORC1 signaling and AMP-activated protein kinase (AMPK) Snf1 is required to induce LD generation at stationary phase in a Dga1-dependent manner. Overnight precultures grown in SC medium were diluted to optical density OD_600_ = 0.15 and allowed to grow for 31–32 h to reach the early stationary phase. BODIPY493/503-associated fluorescence intensity (FI), which is a measure of LD content, was monitored by flow cytometry. For each strain, results are shown as FI fold changes vs. WT. (**C**,**D**) Inactivation of PKA signaling imparted by *PDE2* overexpression, but not the *RAS2*^Val19^ allele (which renders the Ras-PKA pathway constitutively active), promotes LD induction. Cells were grown as indicated, and BODIPY493/503-associated FI was analyzed as in (B). FI ratios between strains are shown for each condition. (**E**) Cells of the specific background were grown overnight in SC medium lacking uracil and leucine in similar conditions as in (B). BODIPY493/503-associated FI was monitored by flow cytometry as in (B). For each strain, results are shown as FI fold changes vs. *SCH9*^WT^. ns—non-significant. Results are mean ± SD of at least three independent experiments. * *p ≤* 0.05; ** *p ≤* 0.01; *** *p ≤* 0.001; **** *p ≤* 0.0001.

Nutrient signaling pathways such as the Target of Rapamycin Complex 1 (TORC1) and Ras-protein kinase A (PKA) play key roles in the regulation of cell growth and physiology. TORC1 is a highly conserved protein kinase that couples changes in amino acids and glucose levels with transcriptional metabolic reprogramming via its downstream effectors, namely Sch9/S6K kinase and Tap42/ PP2A and Sit4/PP6 protein phosphatases [6,7,8,9]. PKA activity is required for cell growth and metabolism and becomes largely inactive in cells deprived of nutrients, contributing to STRE- and Msn2/Msn4-dependent expression of stress response genes and survival at the stationary phase [10]. In response to fluctuations in glucose and amino acid levels, plasma membrane H^+^-ATPase Pma1 and vacuolar ATPase (V-ATPase) coordinately control pH homeostasis. Importantly, V-ATPase modulates Ras-PKA and the TORC1-Sch9 pathways via the GTPases Arf1 and Gtr1, respectively [11,12,13,14]. Interestingly, both PKA and TORC1 also affect the proton-pumping activity of the V-ATPase and conceivably of Pma1 [15]. Some signaling pathways have been recently associated with the regulation of LD metabolism [5]. Importantly, the expression of genes encoding enzymes involved in glycerolipid biosynthesis (Figure 1A) is tightly coordinated with transcriptional responses to changes in choline and inositol levels [16,17,18]. *INO1*, encoding myo-inositol-3-phosphate synthase [19], is the most regulated of the inositol-sensitive upstream activating sequence (UAS*_INO_*) containing genes [16,17,18,20,21], and its expression is sensitive to inositol levels [22]. In response to inositol availability, the expression of *INO1* and other UAS*_INO_* containing genes, namely phospholipid biosynthesis genes, is controlled by the cellular location and activity of the major transcription factor Opi1 [22,23]. In the presence of exogenous inositol, phosphatidylinositol (PI) synthesis is stimulated, phosphatidic acid (PA) levels decrease, and Opi1 repressor is no longer retained in the ER, in part through its interaction with PA [23] (Figure 1A), and binding to the yeast homolog of the integral ER membrane protein VAP (vesicle-associated membrane protein-associated protein) Scs2. Upon activation, Opi1 binds to and represses the Ino2-Ino4 activation complex, which controls UAS*_INO_* required for upregulation of *INO1* and several phospholipid metabolic genes (Figure 1A) [18]. Here we aim to uncover the complex molecular relationships between the activity of major signaling effectors and LD accumulation. We implicate TORC1, PKA, V-ATPase, and Pma1 activities in the regulation of LD metabolism at the stationary phase. We show that *DGA1* expression is metabolically coupled to cell growth in a TORC1-dependent manner, involving downstream effectors Sit4, Mks1, and Sfp1. Furthermore, loss of homeostatic control of cytosolic pH (pHc) due to reduced V-ATPase or Pma1 activities impairs binding of PA to Opi1 and *INO1* expression, thus coupling LD formation to nutrient availability and transcriptional control of lipid biosynthetic genes.

## 2. Results and Discussion

### 2.1. Nutritional Control of LD Formation by TORC1 and PKA

The interdependency of lipid homeostasis with LD metabolism (Figure 1A) is emerging as a hallmark of many diseases, as disruption in lipid processing may contribute to disease progression. Several genome-wide screens have evaluated changes in LD dynamics under specific contexts and treatments [24,25,26,27]. To examine the accumulation of LDs under physiologically-relevant conditions, we established a flow cytometry-based targeted genetic screening of aged cells grown to early stationary phase and labeled with BODIPY493/503. The mutants were firstly tested for growth to discard the nonspecific effect of growth on LD dynamics, and no significant differences were observed at this phase (Figure S1A). To test the feasibility of our screening strategy, we initially tested the effects of abrogation and overexpression of the major TAG synthesizing enzymes Lro1 and Dga1 (Figure 1A). Unlike the *lro1*Δ mutant, *dga1*Δ cells had lower LD content than WT, confirming that Dga1, but not Lro1, was required for NL accumulation at the stationary phase [28]. Importantly, overexpression of *DGA1* enhanced BODIPY493/503-associated fluorescence intensity (FI) signal (Figure 1B). To discard the possibility that variations of BODIPY signal reflect changes in overall ER membrane size, we analyzed the FI signal of an Endoplasmic Reticulum (ER) underexpanded mutant (*ino2*Δ) [29] and an ER overexpanded mutant (*erd1*Δ) [30]. In both cases, we observed an increase in NL content (Figure 1B), indicating that the signal detected reflects alterations in LD content rather than changes in overall ER membrane mass. To further support the feasibility of this approach, we selected mutants with established roles in LD metabolism. As previously described [31], deletion of AMPK *SNF1* enhances LD content, whereas *sit4*Δ cells had the opposite effect. This is consistent with the fact that Snf1 is constitutively activated in the *sit4*Δ strain and inhibits acetyl-CoA carboxylase (Acc1) activity required for FA synthesis (Figure 1A) [31]. Plus, the expression of a constitutively active *ACC1* allele with two site mutations at serines 659 and 1157 (*ACC1*^Ser659Ala,Ser1157Ala^), which are believed to function as phosphorylation sites recognized by Snf1 [32], enhanced NL content in WT but not in the *sit4*Δ mutant (Figure S1B). This essentially phenocopies the *snf1*Δ mutant observations. Remarkably, abrogating Sit4 activity in *snf1*Δ cells restored LD content to levels similar to WT, indicating that the phosphatase also operates independently of Snf1 signaling in the regulation of LD metabolism. Finally, we observed an increase in LD content in *sch9*Δ cells, in contrast to the *sit4*Δ mutant (Figure 1B). This is in agreement with the fact that TORC1 becomes largely inactivated during the stationary phase to block phosphorylation of Sch9 and activate Sit4, thus supporting NL accumulation in LDs [9,33,34,35]. Consistently, TORC1 inhibition by rapamycin in yeast leads to LD synthesis [36] and TORC1 negatively regulates LD metabolism by inhibiting lipin Pah1 [37].

PKA has been implicated in LD dynamics in various organisms [38,39,40], but its role in yeast is poorly understood. We then investigated the contribution of PKA signaling to LD dynamics at the stationary phase using our strategy. Interestingly, constitutive activation of PKA due to the introduction of the dominant-active *RAS2*^Val19^ allele did not alter LD content in WT cells (Figure 1C), whereas expression of cyclic adenosine monophosphate (cAMP) phosphodiesterase *PDE2* (leading to diminished Ras-PKA signaling) raised NL levels in WT cells (Figure 1D). An abrupt glucose deprivation, known to repress PKA activity [13], also induced a transient increase in LD content (Figure S1C). Overall, these results strongly indicate that PKA signaling regulates LD dynamics in yeast.

It was previously shown that the Ras-PKA signaling pathway regulates distinct processes either downstream of TORC1-Sch9 or in a parallel pathway [9]. To establish a relationship between these pathways and differentiate between these possibilities, we expressed either *SCH9*^WT^ or a constitutively active *SCH9*^3E^ [35] (independent of TORC1 activation status) in low (*PDE2* overexpression) and high PKA (*RAS2*^V19^) activity backgrounds. If TORC1-Sch9 is upstream of PKA, we would not expect to see any variation in NL content. In contrast, if TORC1-Sch9 and PKA are involved in LD metabolism in parallel pathways at stationary phase, we would predict that expression of *SCH9*^3E^ would have some inhibitory effect. WT cells expressing SCH9^3E^ did not display any change upon PKA hyperactivation (Figure 1E), whereas LD accumulation imparted by *PDE2* overexpression was partially blocked in cells expressing the mutant *SCH9* allele (Figure 1E). Together, these data argue that TORC1-Sch9 axis can regulate LD metabolism in a pathway upstream or in parallel to Ras-PKA. Nevertheless, we cannot exclude the possibility that PKA and TORC1 may work independently of Sch9 in the regulation of LD metabolism. Collectively, this global screening performed at stationary phase revealed many genes that have previously been described to contribute to LD dynamics (Figure 1B–E), demonstrating the suitability and robustness of this approach. We also provide new insights into the role of major nutrient-sensing TORC1 and PKA signaling pathways in the regulation of LD metabolism at the stationary phase.

### 2.2. TORC1 Controls DGA1 Expression during Growth to Promote LD Generation at Stationary Phase

**Figure 2:**
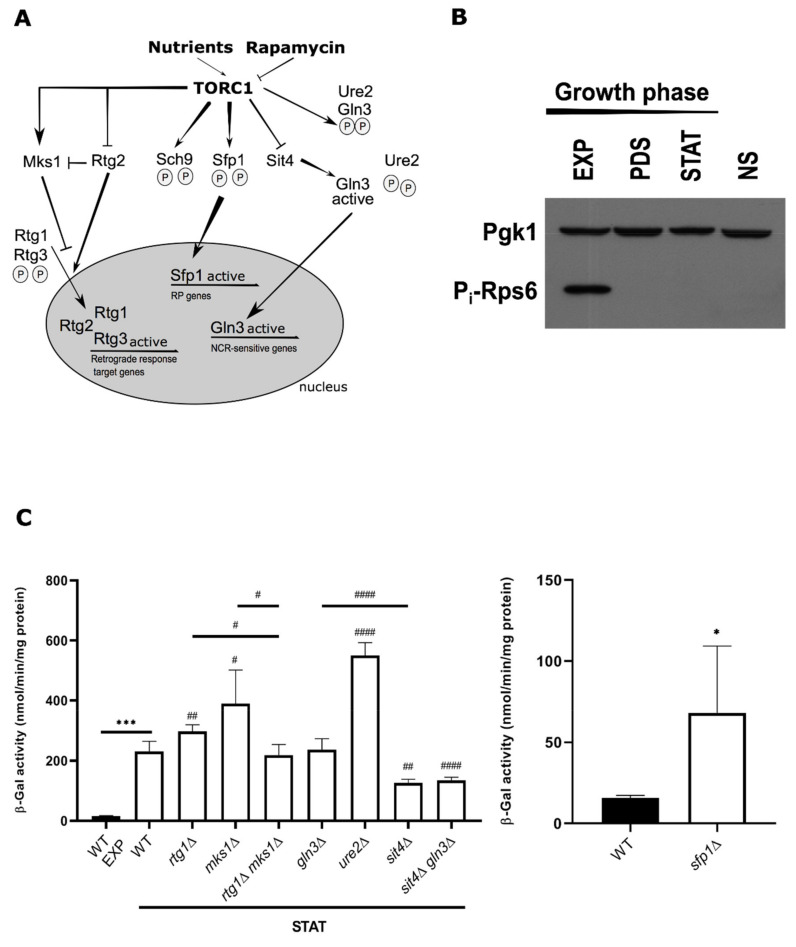
Transcriptional control of *DGA1* expression is coupled to cell growth and is mediated by TORC1 downstream effectors Sit4, Sfp1, and Mks1. (**A**) Overview of TORC1-controlled transcriptional program. (**B**) Western blot showing TORC1 activity changes for the indicated periods of growth, as measured by the phosphorylation of *bona fide* TORC1 target ribosomal protein S6 (Rps6). Pgk1 was used as a loading control. WT cells were grown in SC medium during growth, and aliquots were collected at different growth stages. For the nitrogen starvation experiment (used as internal control), cells were grown in SC medium to exponential phase and shifted to nitrogen-free medium for 90 min. EXP—exponential; PDS—post-diauxic shift; STAT—stationary; NS—nitrogen starvation. (**C**) *DGA1* expression is controlled by TORC1-regulated targets Sit4, Mks1, and Sfp1. *DGA1* promoter was cloned into the LacZ reporter plasmid YEp357 as described in Section 3 and transformed into the indicated strains. Overnight precultures grown in SC medium lacking uracil were diluted and allowed to grow to reach early stationary phase (left panel, as in Figure 1B), or to exponential phase (right panel). Protein extracts were prepared, and specific β-galactosidase (β-Gal) activities, with o-nitrophenyl-β-D-galactopyranoside (ONPG) as a substrate, were determined by measuring the amount of o-nitrophenol released by the galactosidase-catalyzed hydrolysis process. Results are mean ± SD of at least three independent experiments. *^,#^ *p* ≤ 0.05; ^##^ *p* ≤ 0.01;*** *p* ≤ 0.001; ^####^ *p* ≤ 0.0001.

It is known that TORC1 controls numerous aspects of gene transcription in eukaryotes in response to several metabolic and environmental cues (Figure 2A). The TOR pathway is responsible for setting the steady-state level of gene expression, and rapamycin has immediate, profound effects on gene transcription and ribosome biogenesis, even before adaptation to fluctuations in nutrient signaling [41]. To define novel mechanistic functions carried out by TORC1 in LD dynamics, we first sought to determine if TORC1 regulates *DGA1* expression in response to TORC1 inhibitor rapamycin, using a *DGA1*-LacZ transcriptional fusion reporter. We observed a slight but consistent increase of *DGA1*-LacZ transcriptional activity upon rapamycin treatment, indicating that TORC1 represses *DGA1* expression (Figure S2A). We then investigated if we could observe a similar increment in *DGA1* transcriptional activity upon transition from exponential (EXP, nutrient-rich conditions) to the stationary phase (STAT, acute nutrient starvation). Our results show that, as cells progress from EXP to post-diauxic (PDS) and STAT phase, TORC1 downregulation (Figure 2B) is accompanied by an approximately 15-fold increase in *DGA1* promoter activity (Figure 2C, left panel). Overall, reduced TORC1 signaling seems to be correlated with the stimulation of *DGA1* transcription at the stationary phase. In agreement, *sit4*Δ cells displayed ~45% reduction in transcriptional activity of *DGA1* promoter (Figure 2C, left panel), and this is associated with lower LD content at stationary phase (Figure 1B). Therefore, Sit4 links TORC1 signaling to LD metabolism by targeting *DGA1* expression during the stationary phase.

To address how TORC1 downregulation might connect *DGA1* expression to LD accumulation, we used the bioinformatics screening tool YEASTRACT+ [42] to find potential transcription factors (TF) regulating *DGA1* transcription. Inspection of the promoter region of the *DGA1* gene revealed the presence of several response elements for numerous TFs (Table S1A,B). We focused on Gln3, Rtg1/Rtg3, and Sfp1 TFs, which were selected based on the following criteria: (1) TFs regulated by *bona fide* nutrient and stress signaling proteins TORC1, Sch9, or Sit4, or (2) TFs predicted to be active at stationary phase to work as potential positive regulators of *DGA1* expression or (3) TFs active at exponential phase (under nutrient rich-conditions, where TORC1 is active) that may act as negative regulators of *DGA1* expression.

Gln3 is a GATA transcriptional activator in the nitrogen catabolite repression system, regulated by nitrogen sources and Ure2. When TORC1 becomes inactive, Sit4 dephosphorylates Gln3, which dissociates from Ure2 and relocates to the nucleus to activate transcription (Figure 2A) [43,44,45,46,47,48]. We observed that deletion of *GLN3* in WT or *sit4*Δ cells had no effect on the reporter expression, but the loss of Ure2, where Gln3 is constitutively nuclear [44,49], stimulated *DGA1* promoter activity (Figure 2C, left panel). This indicates that Ure2-dependent import of Gln3 into the nucleus [46] is required for *DGA1* transcription at the stationary phase. Notably, abolishing Sit4 activity decreased *DGA1*-LacZ activity by ~40–50% in the *gln3*Δ strain, indicating that Sit4 works independently of Gln3 in the regulation of *DGA1* transcription. Altogether, we establish that Sit4 stimulates *DGA1* expression (Figure 2C, left panel) and favors Acc1 activation (Figure S1B) required for acyl-CoA generation by inhibiting Snf1 to collectively promote LD generation at the stationary phase (Figure 1B).

Rtg1 and Rtg3 are both basic, helix-loop-helix TFs (bHLH-LeuZip) that require each other to perform their function in a heterodimeric complex in the retrograde (RTG) pathway. Translocation of Rtg1-Rtg3 to the nucleus requires Rtg2, which is negatively controlled by TORC1 via Lst8. Mks1 acts in concert with TORC1 (Figure 2A) to negatively regulate retrograde signaling by forming a complex with either Bmh1 or Bmh2, which maintains Rtg3 in a hyperphosphorylated state [50,51]. Our results show little change in *DGA1* expression upon *RTG1* deletion, however, in *mks1*Δ cells, where the RTG pathway is constitutively active, *DGA1* expression increased in an Rtg1-dependent manner (Figure 2C, left panel). This indicates that decreased TORC1 signaling favors *DGA1* expression by downregulating Mks1 inhibitory activity over Rtg1 during the stationary phase.

Here, we found differences in the effect of Sit4 and Mks1 upon rapamycin treatment and at the stationary phase (Figure S2A,C, left panel). Since this was a short-term rapamycin treatment (2 h treatment under nutrient-rich conditions), we consider that this does not recapitulate the conditions to fully induce *DGA1* expression as observed at the stationary phase (upon acute nutrient deprivation). In fact, the transition from exponential to stationary phase is correlated with a much larger increase in *DGA1* expression (15-fold increase) than the one observed in dimethyl sulfoxide (DMSO) versus rapamycin-treated cells (1.6-fold increase). The fact that we do not observe similar effects on *sit4*Δ and *mks1*Δ mutants indicates that these proteins likely integrate a complex signaling circuit that goes beyond regulation by TORC1 at the stationary phase. The Ras-PKA pathway, which is largely inactive during the stationary phase and is associated with alterations in NL content (Figure 1C–E), may well impact this context as both TORC1 and PKA pathways share common targets to control transcription [41,52,53]. Finally, specific induction of *DGA1* expression is particularly noticeable in the *sfp1*Δ mutant with altered ribosomal protein (RP) expression and ribosome biogenesis [54], which in turn is consistent with the strong impact of rapamycin on gene expression [7,9]. Indeed, this mutant displayed increased *DGA1* expression at both basal (Figure 2C, right panel) and inducible (rapamycin-treated cells) conditions (Figure S2A), indicating that Sfp1 negatively regulates *DGA1* expression under nutrient-rich conditions (Figure 2C, right panel). Previous Chromatin Immunoprecipitation sequencing (ChIP-seq) experiments support that changes in Sfp1 levels affect *DGA1* transcription, although no evidence of direct Sfp1 binding to *DGA1* promoter was observed [55]. In summary, our results indicate that TORC1 downstream effectors Sit4, Mks1, and Sfp1 regulate *DGA1* transcription at stationary phase.

### 2.3. Pma1 and V-ATPase Activities Control LD Accumulation

**Figure 3:**
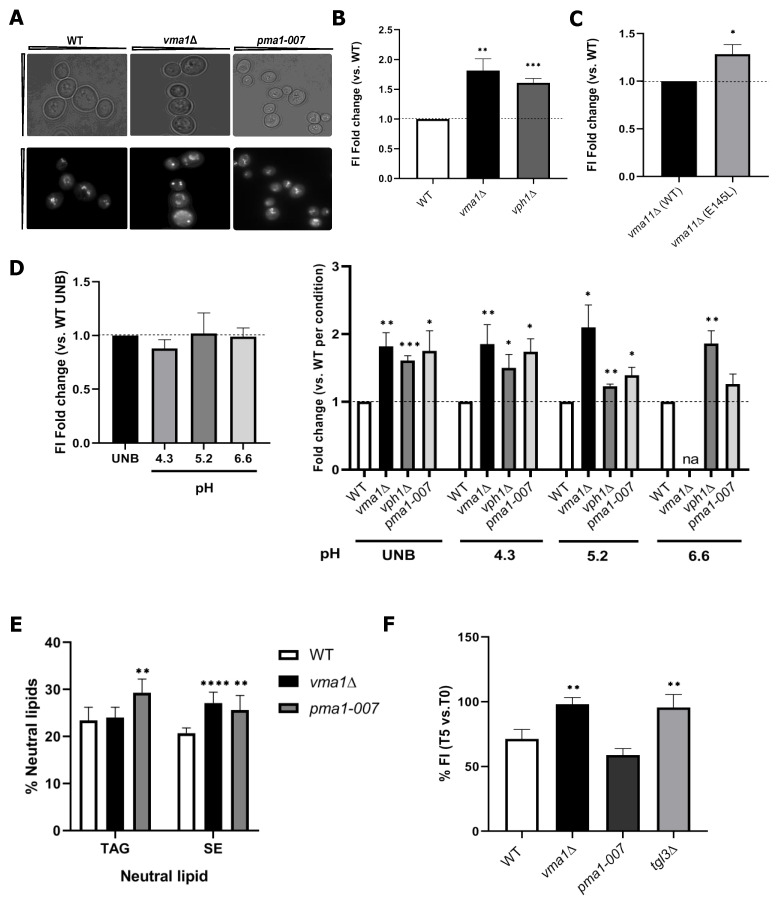
Deregulation of intracellular pH due to loss of V-ATPase and Pma1 activities is associated with perturbed LD metabolism. (**A**) LD staining of WT, *vma1*Δ, and *pma1-007* cells with BODIPY493/503 and analysis by fluorescence microscopy. Cells were grown as described in Figure 1B. Scale bar, 2 µm. (B and C) The *vph1*Δ mutant, and *vma11*Δ cells carrying *VMA11* WT or the *VMA11*^E145L^ mutation, have higher NL content. Growth conditions and BODIPY493/503-associated FI measurements are described in Figure 1B. For each strain, shown are FI fold changes vs. WT (**B**), or normalized to *vma11*Δ cells expressing *VMA11* WT allele (**C**). (**D**) Intracellular acidification upon deletion of *VMA1* or *VPH1*, and *pma1-007* mutation leads to higher LD induction, irrespective of extracellular pH. Cells were grown in SC unbuffered (UNB) or buffered to low, medium, and high pH (pH 4.3, 5.2, and 6.6, respectively) media, as described in (A), and fluorescence intensity analysis by flow cytometry is described in (B). For each condition, shown are FI fold changes vs. WT UNB. na—non-applicable. (**E**) V-ATPase and *pma1-007* mutants have increased neutral lipid content. Cells of indicated genotypes were grown overnight in SC medium, diluted in SC medium containing [^3^H]acetate, and grown for 31–32 h to early stationary phase. Lipids were extracted and separated by TLC. TAG- and SE- to-total lipid ratio is plotted and represented as a percentage of neutral lipid. TAG—triacylglycerol; SE—sterol esters. (**F**) Increased LD content upon loss of V-ATPase activity (*vma1*Δ) is associated with reduced LD consumption by lipolysis. LD consumption was followed in cells with the indicated genotype grown in SC media with cerulenin (10 µg/mL). At time 0 (T_0_) and after 5 h (T_5_) of growth resumption, LD content was examined by monitoring the variation in BODIPY493/503-associated FI, as described in (B). Results are mean ± SD of at least three independent experiments. * *p ≤* 0.05; ** *p ≤* 0.01; *** *p ≤* 0.001; **** *p ≤* 0.0001.

Alterations in V-ATPase activity and lysosomal pH dysregulation have been heavily implicated in aging [56] and adult-onset neurodegenerative diseases [57]. More importantly, variations in LD metabolism are now increasingly recognized in some groups of neurodegenerative diseases [4]. In our screening, *VMA1*, encoding for the catalytic subunit A of the peripheral V1 complex of V-ATPase, has emerged as a potential negative regulator of LD metabolism (Figure 1B). To better understand the role of V-ATPase in NL storage, we firstly observed *vma1*Δ cells stained with BODIPY493/503 by fluorescence microscopy (Figure 3A) and performed LD number quantification (as defined by the LD index, see Section 3). We observed that higher LD content detected in our screening (Figure 1B) is associated with an increase in LD number in the V-ATPase mutant (Figure S2B). To confirm that this is a general effect imparted by the loss of V-ATPase activity, we independently tested the *vph1*Δ mutant, which has a comparable level of V-ATPase activity observed in *vma* mutants, and the *VMA11*^E145L^ mutation, that allows V-ATPase assembly but abolishes both ATPase activity and proton transport [58,59]. Both strains exhibited higher NL content, showing that V-ATPase activity is coupled to LD metabolism at the stationary phase (Figure 3B,C). V-ATPase mutants have lower pHc, and are unable to properly respond to glucose replenishment [11,60]. Given that glucose deprivation promotes a reduction in pHc [11,60,61], and this was associated with a transient increase in LD content (Figure S1C), we were intrigued by the idea that pHc could be the missing link between V-ATPase function and LD accumulation. We then decided to investigate if the increase in LD content upon loss of V-ATPase activity could be associated with fluctuations in pHc. For that, WT, *vma1*Δ and *vph1*Δ cells were grown in media buffered at different extracellular pHs (pHext), and checked the effect on LD content. First, we analyzed BODIPY493/503-associated FI in cells grown in SC unbuffered (UNB) or buffered to low, medium and high pH (pH 4.3, 5.2 and 6.6, respectively). Whereas LD levels remained unaltered in WT cells (Figure 3D, left panel), there was a ~2 fold-increase in LD content in the *vma1*Δ mutant, irrespective of the extracellular pH (Figure 3D, right panel). The *vph1*Δ mutant, which supports high pH (pH 6.6), also displayed an LD accumulation phenotype. These results suggest that perturbed pHc affects LD homeostasis. To reinforce this association, we analyzed LD content in WT cells shifted to SC medium buffered to low and high pH, and supplemented with the proton ionophore carbonyl cyanide 3-chlorophenylhydrazone (CCCP), which disrupts the plasma membrane proton gradient and blocks the ability to regulate pHc [62]. In both conditions, we observed a ~50% increase in FI signal (Figure S2C), suggesting that Pma1 control over pHc also contributes to LD dynamics. Next, we decided to test the effect of physiological changes in pHc on cells with reduced levels of Pma1, using a genetic approach. Acute and chronic loss of V-ATPase activity promotes the internalization of ~50% of surface Pma1, a comparable reduction in *PMA1* expression observed in a *pma1-007* mutant. This results in a diminished capacity to pump protons out of the cell with a concomitant reduction in cytosolic pHc [60,63,64]. The results revealed an increase in the BODIPY493/503-associated FI signal in *pma1-007* mutant at all pHext values (Figure 3D, right panel), and this was correlated with higher LD number, as observed for *vma1*Δ cells (Figure 3A and Figure S2B). To validate changes in NL content observed in *vma1*Δ and *pma1-007* mutants using our approach, we performed thin-layer chromatography (TLC) analysis for quantification of SE and TAG levels. We observed that *vma1*Δ cells specifically accumulated SEs, whereas TAG levels remained constant, a trend observed for other *vma* mutants [65]. The *pma1-007* mutant showed increased levels of both TAG and SE (Figure 3E), thus confirming our previous findings (Figure 3D). Altogether, these data strongly support that the rapid drop in pHc (either by glucose deprivation, external treatment with CCCP or genetically by abolishing V-ATPase or reducing Pma1 levels) is sufficient to prompt LD formation.

**Figure 4:**
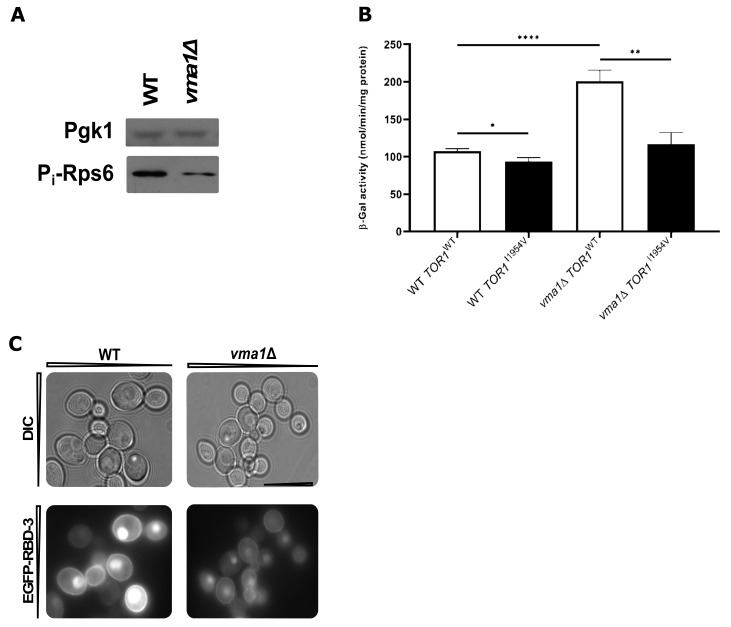
Loss of V-ATPase activity leads to lower TORC1 and Ras-PKA activation. (**A**) Western blot showing TORC1 activity changes in WT and *vma1*Δ cells, as measured by the phosphorylation of Rps6. Pgk1 was used as a loading control. WT cells were grown in SC medium to the exponential phase, and aliquots were collected and processed for Western blotting. (**B**) WT or *vma1*Δ cells expressing either WT or a hyperactive *TOR1* allele (*TOR1*^I1954V^) and YEp357-*DGA1*-LacZ reporter gene were grown in SC medium lacking uracil and leucine to early stationary phase. Protein extracts were prepared, and specific β-Gal activities, with ONPG as a substrate, were determined by measuring the amount of o-nitrophenol released by the galactosidase-catalyzed hydrolysis process (as in Figure 2C). Results are mean ± SD of at least three independent experiments. * *p* ≤ 0.05; ** *p* ≤ 0.01; **** *p* ≤ 0.0001. (**C**) RBD-GFP localization was inspected in WT and *vma1*Δ cells grown in SC medium lacking uracil to the exponential phase. Scale bar, 2 µm.

The observed LD accumulation phenotype in *vma1*Δ and *pma1-007* strains could also arise from impaired lipolysis, through which TAG is hydrolyzed back to glycerol and FAs by TAG lipases (Tgl proteins). To test this hypothesis, WT, *vma1*Δ, *pma1-007,* and *tgl3*Δ cells (lacking the major TAG lipase) were grown to stationary phase and diluted into a fresh medium containing the FA biosynthesis inhibitor cerulenin (Figure 3F), a condition known to promote lipolysis. After 5 h of growth resumption, LD content was examined. Unlike WT cells, the *vma1*Δ mutant had decreased LD consumption to a level comparable to *tgl3*Δ cells (Figure 3F). However, *pma1-007* mutation displayed WT kinetics, indicating that pHc per se is not a general feature affecting LD utilization, but rather the loss of V-ATPase activity and changes in downstream signaling likely contributes to aberrant LD dynamics (Figure 1B–D). Ras-PKA and TORC1 are both activated through the V-ATPase, which in turn works as a sensor for pHc in vivo [11,13,14]. Based on our observation that reduced PKA and TORC1 activities lead to an increase in NL levels, we hypothesize that altered TORC1 and Ras-PKA signaling may be contributing factors to LD accumulation in *vma1*Δ cells. To test alterations in the activation of these signaling pathways in the mutant, we monitored TORC1 and Ras-PKA activities. At the exponential phase, we observed lower TORC1 activation in *vma1*Δ cells, as monitored by Rps6 phosphorylation (Figure 4A). It is then possible that *DGA1* expression may be disturbed in a TORC1-dependent manner in this mutant. To test this hypothesis, we used WT and *vma1*Δ cells carrying *DGA1*-LacZ and introduced plasmids expressing either WT or a hyperactive *TOR1* allele (*TOR1*^I1954V^) [66]. We observed a subtle but consistent decrease in β-Gal activity in WT cells with the *TOR1*^I1954V^ mutation (Figure 4B). This result corroborates with the opposite effect observed in WT rapamycin-treated cells (Figure S2A) and strongly indicates that TORC1 represses *DGA1* expression. Importantly, *DGA1* expression is increased in the *vma1*Δ mutant when compared to WT cells (Figure 4B), and expression of *TOR1*^I1954V^ leads to a significant reduction in *DGA1*-LacZ reporter activity in these cells (Figure 4B). Hence, this increase in *DGA1* expression is a result of altered TORC1 activity in *vma1*Δ cells. Dga1 is the main DGAT responsible for TAG synthesis at the stationary phase, as shown by others and corroborated by our screening approach, but no changes in TAG levels were observed in the mutant (Figure 3E). In this case, we believe that the expression and/or activity of Are1 and Are2 might be altered and actually be responsible for channeling fatty acids to ergosterol to generate SE in the mutant, whose levels are increased (Figure 3E). Fei et al. [65] have reported that a significant increase in the number of LDs observed in *vma9*Δ cells was associated with significant upregulation of ASAT activity and enhanced SE levels. Importantly, this increase in SE was specific since no change in TAG levels was detected in *vma9*Δ cells when compared to WT cells. Overall, loss of V-ATPase activity seems to have unanticipated effects on SE storage (Figure 3E). Because of this, it is possible that despite increased *DGA1* expression, posttranslational modifications might affect Dga1 stability, and/or activity in *vma1*Δ cells.

To evaluate the activation of the Ras-PKA signaling pathway, we made use of the GFP-RBD probe, which allows the assessment of activated Ras [67]. At the exponential phase, we observed that the probe was localized to the plasma membrane and within the nucleus in WT cells (Figure 4C), as reported [67]. Loss of V-ATPase activity imparted by the deletion of *VMA1* leads to a substantial decrease of Ras activity, as the GFP-RBD signal appeared weaker, more diffuse, and evenly distributed within the cells (Figure 4C). Altogether, our work provides an additional line of evidence that V-ATPase links pHc to Ras-PKA activation and LD dynamics and that failure to regulate pHc leads to aberrant NL accumulation. In agreement with this idea, overexpression of *PDE2* had no effect in LD content in *vma1*Δ cells, in contrast to WT cells (Figure 1D), indicating that inhibition of PKA signaling requires a functional V-ATPase to promote LD accumulation. Moreover, hyperactivation of PKA abrogates LD formation in *vma1*Δ cells (Figure 1C), suggesting that inhibition of PKA downstream of V-ATPase (Figure 4C) contributes, at least in part, to abnormal NL levels. Importantly, as shown in this study, glucose deprivation (Figure S1C), which is known to promote V-ATPase disassembly concomitant with a reduction of pHc and downregulation of PKA signaling, also promotes transient LD accumulation [11,13]. Collectively, our results are consistent with a model in which PKA controls glucose-dependent V-ATPase assembly to raise cytosolic pH and inhibit NL accumulation (Figure 1C and Figure S1C). In addition, cytosolic pH functions as a second messenger via V-ATPase, to regulate PKA activity and LD accumulation (Figure 1C,D and Figure 4C). Overall, our findings demonstrate for the first time that the change in intracellular pH serves as a major metabolic signal to regulate LD generation, with foreseeable consequences for cellular dysfunction and aging when control of pHc is disrupted.

### 2.4. V-ATPase Function Deficiency Confers Inositol Auxotrophy Associated with Altered Opi1 Localization and Activity

**Figure 5:**
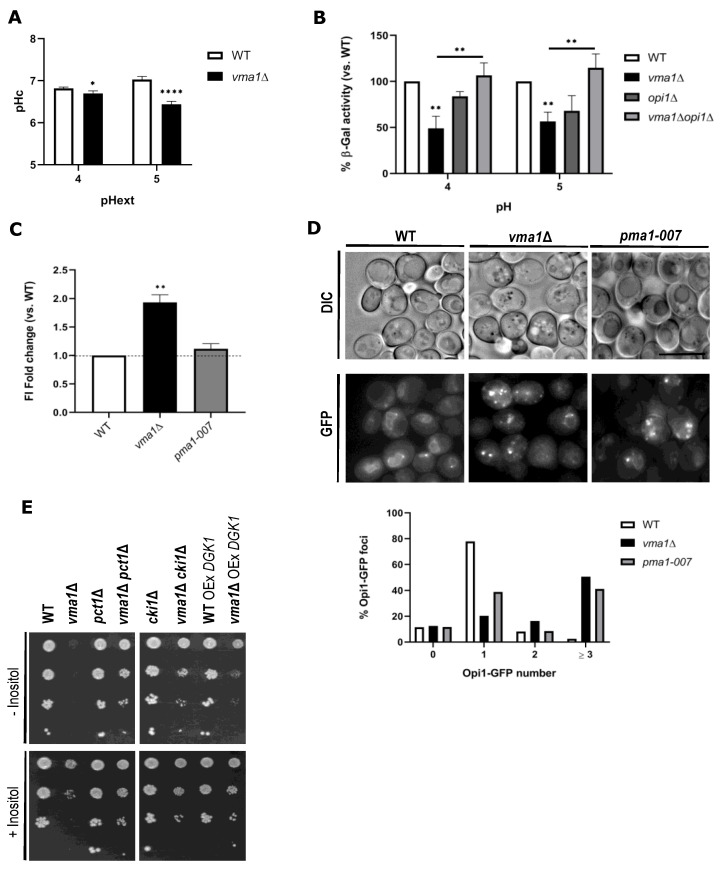
Cytosolic acidification leads to alterations in Opi1 transcriptional repressor activity and localization, thus contributing to aberrant lipid metabolism and inositol auxotrophy. (**A**) Cells devoid of *VMA1* display cytosolic acidification. pHc was measured with a pH-sensitive GFP probe (pHluorin) as described in Section 3. Cells were grown to an exponential phase in SC media buffered at the indicated pH. (**B**) Opi1-mediated repression of *INO1* promoter activity in *vma1*Δ cells. Overnight precultures grown in SC medium lacking inositol and leucine were diluted and allowed to grow to reach an exponential phase in fresh media buffered at the indicated pH. Protein extracts were prepared, and specific β-Gal activities were determined as in Figure 2C. Results are shown as a percentage of WT levels. (**C**) Higher Opi1 repressor activity is associated with higher Opi1-GFP levels in *vma1*Δ cells. WT, *vma1*Δ, and *pma-1007* cells expressing Opi1-GFP were grown in SC medium lacking leucine, as described in Figure 1B, and fluorescence intensity analysis of the GFP signal was performed by flow cytometry (FL1 channel). For each strain, shown are FI fold changes vs. WT. (**D**) Altered intracellular pH affects Opi1 localization in *vma1*Δ and *pma-1007* strains. WT, *vma1*Δ, and *pma-1007* cells expressing Opi1-GFP were grown in SC medium to exponential phase. Opi1-GFP localization was monitored by fluorescence microscopy (higher panel) and quantification of the number of Opi1-GFP puncta is defined as a percentage of Opi1-GFP foci (*n* > 100 cells; lower panel). Scale bar, 2 µm. DIC—Differential Interference Contrast; GFP—Green Fluorescent Protein. (**E**) Inactivation of the CDP-choline pathway or *DGK1* expression suppresses the inositol auxotrophy in *vma1*Δ cells. Cells of indicated genotypes were grown in SC medium lacking inositol to the exponential phase and diluted to OD_600_ = 0.1. Tenfold dilution series were spotted onto buffered SC plates (pH 5) without or supplemented with inositol and incubated at 26 °C. Results are mean ± SD of at least three independent experiments. * *p ≤* 0.05; ** *p ≤* 0.01; **** *p ≤* 0.0001.

There is a physiological connection between pHc and the regulation of phospholipid metabolism [60]. A rapid decrease in intracellular pH impairs the electrostatic interaction between PA to Opi1, thus increasing its transcriptional repression of phospholipid metabolic genes under the control of the Ino2/Ino4 complex [60]. Notably, Opi1-dependent repression of UAS_INO_ at lower pHc values in the *pma1-007* mutant and during Pma1 inhibitor ebselen treatment was reported [60]. Based on our work, we anticipate that repression of Opi1-regulated phospholipid synthesis genes leads to the accumulation of lipid precursors upstream of cytidine diphosphate diacylglycerol (CDP-DAG), thus favoring LD generation in the *pma1-007* mutant (Figure 3D,E). Consistent with this hypothesis, *INO2* deletion, which phenocopies Opi1 repression, enhanced NL content (Figure 1B). To better understand how V-ATPase might link pHc to LD generation, we first assessed pHc values in *vma1*Δ mutant at different pHext. The results show that pHc is lower in *vma1*Δ cells, confirming an acidified cytosol (Figure 5A). To evaluate whether cytosolic acidification causes derepression of Opi1 activity, we measured transcription of Opi1-dependent genes using an *INO1*-LacZ transcriptional reporter in the *vma1*Δ strain. At both pH 4 and pH 5, we found almost ~40–50% repression, which was restored to WT levels by deletion of *OPI1* (Figure 5B). To verify if this repression was associated with changes in *OPI1* expression, we monitored Opi1-GFP levels in WT and *vma1*Δ mutant. Our results show a 2-fold increase in *vma1*Δ cells but no change in *pma1-007* cells (Figure 5C). Overall, these results reinforce our hypothesis that Opi1 could turn on LD production by inhibiting phospholipid biosynthetic genes expression, thereby stimulating NL synthesis and LD generation when V-ATPase and Pma1 control of pHc is lost.

Since pHc affects Opi1-PA interaction and we observed transcriptional repression of *INO1* in *vma1*Δ cells, we decided to monitor the localization of Opi1-GFP in WT and *vma1*Δ cells. In WT cells, we detected perinuclear localization of Opi1-GFP, mostly with 1 punctum (Figure 5D), at exponential phase. Strikingly, Opi1-GFP became more diffuse, and we observed multiple foci in *vma1*Δ cells (>2–3 foci), likely because PA is now poorly recognized by Opi1 in an acidified cytosol (Figure 5D). Similar changes were observed in *pma1-007* mutant, with most cells with either 1 or more than 3 foci. A similar distribution was also reported for Opi1^FFAT^-GFP labeled puncta, close to the perinuclear ER, which was related to possible pools of PA associated with nascent LD formation sites [18]. Importantly, alterations in Opi1 localization in *vma1*Δ cells are not associated with changes in PA levels, as a shift to inositol free medium in the presence of choline, a condition known to rise PA levels [18], did not change the extent of Opi1-related transcriptional repression in the mutant (Figure S3B and Figure 5B).

Similarly to Opi1, mammalian IP6K1 protein was reported to bind to PA in the cytosol and to translocate into the nucleus to negatively regulate *INO1* transcription [68]. This led us to investigate if IP6K1 could fulfill Opi1 function in yeast, particularly in the regulation of inositol metabolism upon loss of V-ATPase activity. Bioinformatics analysis of the Bayesian phylogenetic tree revealed that Opi1 sequences are represented as a sister group relative to higher eukaryote IP6K1 sequences (Figure S2D). Importantly, repression of *INO1*-LacZ is associated with an inositol auxotrophy phenotype in *vma1*Δ cells, which was rescued by *OPI1* deletion (Figure 5E and Figure S3A). Expression of WT IP6K1 or the corresponding kinase-dead IP6K1 KD version failed to re-establish the inositol auxotrophy phenotype in *vma1*Δ*opi1*Δ cells, as observed when Opi1-GFP was re-expressed in the double mutant (Figure S3A). This indicates that IP6K1 cannot fulfill Opi1 function, albeit being involved in the regulation of *INO1* transcription and inositol metabolism (Figure S3A). Overall, these data indicate that pHc controls the metabolic switch between phospholipid synthesis and lipid storage by affecting Opi1 localization and repressor activity.

### 2.5. Deregulation of NL Homeostasis Is Associated with Abnormal Phospholipid Metabolism in vma1Δ Cells

Given that NL levels are enhanced in the *vma1*Δ mutant, and this correlates with higher Opi1 repressor activity, we anticipate alterations in phospholipid metabolism associated with the inositol-dependent phenotype. Intriguingly, inactivation of *OPI1* did not fully suppress inositol auxotrophy of the *vma1*Δ mutant to the same extent as inositol supplementation (Figure S3A), supporting the idea that transcriptional repression of *INO1* is not the sole contributing factor. This led us to further investigate if inositol auxotrophy could also be attributed to aberrant lipid metabolism. Overexpression of diacylglycerol kinase *DGK1*, which catalyzes the conversion of DAG to PA, improved the capacity of *vma1*Δ cells to grow in media lacking inositol (Figure 5E). This result raises the possibility that this phenotype may arise from CDP-choline pathway hyperactivity derived from increased DAG pools. As phospholipid biosynthetic pathway is impaired in *vma1*Δ strain, we postulate that the CDP-choline pathway may represent a major metabolic sink for excess DAG, while working as an alternative pathway to support phosphatidylcholine (PC) biosynthesis, independently of the CDP-DAG pathway. This would explain why PC levels are unchanged in *vma1*Δ cells [69], despite the fact that this pathway is transcriptionally repressed by Opi1. A prediction of this model is that genetic inactivation of the CDP-choline branch should also suppress inositol auxotrophy of *vma1*Δ cells. In fact, either deletion of choline kinase *CKI1* or cholinephosphate cytidylyltransferase *PCT1* improves the ability to grow on inositol-free plates (Figure 5E). In summary, we show for the first time that the physiological basis for *vma1*Δ-associated inositol auxotrophy is related to abnormal lipid metabolism, apart from defects in *INO1* transcription. Consistent with atypical phospholipid metabolism, a previous study reported lower PI levels in the absence of inositol, and lower phosphatidylethanolamine (PE) and phosphatidylserine (PS) content in *vma1*Δ cells. Importantly, this was associated with lower PS decarboxylase activity in the mutant [69]. Since *PSD1* expression is under the control of the Ino2-Ino4 activator complex [70] and regulates phospholipid synthesis downstream of PE [71], this PE unbalance is seemingly related to transcriptional repression of *PDS1* and other biosynthetic genes controlled by Opi1, as shown in this study.

### 2.6. Uncoupling Opi1 Activity from PA Binding Is Sufficient to Induce Inositol-Dependent Phenotypes and LD Generation

**Figure 6:**
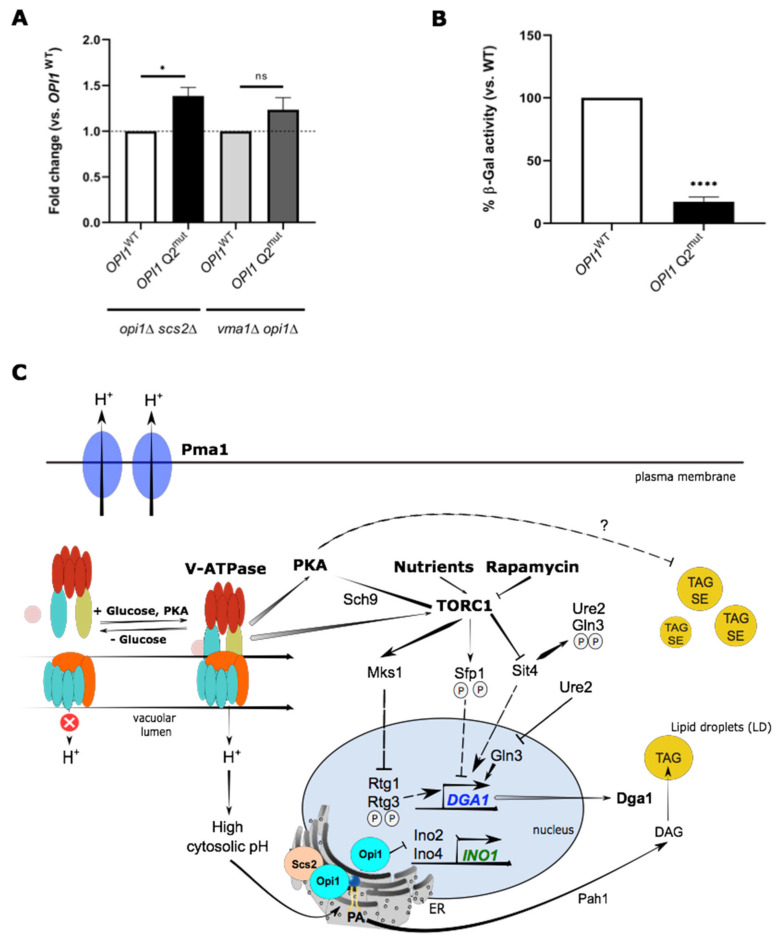
The decreasing affinity of Opi1 to PA is sufficient to stimulate LD generation associated with Opi1-mediated repression of the *INO1* promoter. (**A**) Expression of a mutant in the Opi1 Q2 domain (Q2^mut^), which decreases affinity toward PA, induced LD production. *opi1*Δ*scs2*Δ and *vma1*Δ*opi1*Δ cells expressing *OPI1*^WT^ or *OPI1*-Q2^mut^ were grown in SC medium lacking uracil, as described in Figure 1B, and BODIPY493/503-associated fluorescence intensity (FI) was monitored by flow cytometry. For each strain, shown are FI fold changes vs. strain expressing *OPI1*^WT^. (**B**) Expression of *OPI1*-Q2^mut^ increased Opi1-mediated repressor activity over the *INO1* promoter. *opi1*Δ*scs2*Δ cells expressing *OPI1*^WT^ or *OPI1*-Q2^mut^, and *INO1*-LacZ, were grown to exponential phase in SC medium lacking uracil, leucine, and inositol. Protein extracts were prepared, and specific β-Gal activities were determined as in Figure 2C and normalized to *opi1*Δ*scs2*Δ cells expressing *OPI1*^WT^ levels. (**C**) TORC1, PKA, V-ATPase, and Pma1 integrate a complex signaling network to regulate LD metabolism. V-ATPase and Pma1 tightly control cytosolic pH, which affects the electrostatic interaction and affinity of Opi1 towards PA in the ER. This, in turn, alters Opi1 localization and attenuates transcriptional activation of *INO1* and genes of the phospholipid biosynthetic pathway by the Ino2/Ino4 complex. In response to nutrients and growth status, TORC1 regulates Sit4, Mks1, and Sfp1 activities for transcriptional control of *DGA1*. PKA and is responsive to cytosolic pH via V-ATPase and modulates glucose-dependent V-ATPase assembly to raise cytosolic pH and inhibit NL accumulation. P—phosphorylation. Results are mean ± SD of at least three independent experiments. * *p ≤* 0.05; **** *p ≤* 0.0001. ns—non-significant.

Our results thus far are consistent with a model where acidification of the cytosol affects the electrostatic binding of Opi1 to PA and leads to Opi1-mediated repression of the phospholipid biosynthetic pathway, thus shunting lipid precursors towards NL synthesis and LD formation. These findings raised the question of whether PA-mediated sequestration of Opi1 directly influences lipid storage. To test this, we untethered Opi1 from the ER by removing *SCS2*, such that Opi1 activity relies mainly on its ability to bind PA via its Q2 domain. Expression of the Opi1 mutant Q2 domain (Q2^mut^), which has lower affinity toward PA [72], stimulated LD generation in *opi1*Δ*scs2*Δ cells (Figure 6A), and this was correlated with lower LacZ activity (Figure 6B). These results essentially recapitulate *vma1*Δ phenotypes (Figure 1B, Figure 3B and Figure 5A,B). Importantly, expression of Q2^mut^ in the *vma1*Δ*opi1*Δ mutant had no effect on LD content, where Opi1-PA interaction is expected to be weakened (Figure 6A). These data collectively indicate that pHc downstream of V-ATPase and Pma1 controls inositol metabolism and membrane biogenesis. Collectively, we demonstrate that a decrease of pHc weakens the interaction between PA and Opi1, leading to higher repressor activity over *INO1* transcription (and overall phospholipid biosynthetic pathways genes) under the control of Ino2/Ino4 complex. Opi1 has been shown to be a substrate for PKA phosphorylation, which enhances its activity as a repressor of *INO1* [73]. Moreover, it was previously demonstrated that a hyperactive variant of the TORC1 kinase complex (*TOR1*^L2134M^ allele) reduced the expression of *INO1* [74]. Based on the results reported in this manuscript, we posit that weakened electrostatic interaction of Opi1 with PA promoted by decreased cytosolic pH is sufficient to favor Opi1 repressor activity and reduce *INO1* transcription (Figure 6A), and confer an inositol auxotrophy phenotype (Figure 5E), thus leading to LD dyshomeostasis (Figure 6B). This would bypass any regulation of Opi1 by TORC1 and PKA complexes, even in circumstances where Opi1-mediated *INO1* transcription would be stimulated rather than repressed, considering that the activity of PKA and TORC1 signaling complexes are reduced at a steady state in the *vma1*Δ mutant (Figure 4A,C). We thus conclude that, under conditions of reduced pHc, Opi1 displays reduced affinity toward PA, and inactivation of PKA and TORC1 pathways relays signals to additional regulatory proteins other than Opi1 to modify LD metabolism upon loss of V-ATPase activity. Among those proteins, lipin Pah1 would be a suitable candidate. It was previously shown that phosphorylation by PKA has a strong inhibitory effect on Pah1 activity, affects its cellular location, and protects it from proteasome-mediated degradation [75,76,77]. TORC1 negatively regulates LD metabolism by inhibiting Pah1 [37]. Whether PKA and TORC1 act through Pah1 in the context of loss of pH homeostasis should be explored in future studies.

## 3. Materials and Methods

### 3.1. Yeast Strains and Plasmids

Yeast strains and plasmids used in this study are listed in Table S2. The strains used were isogenic to BY4741 (MATa *ura3*Δ0 *his3*Δ1 *leu2*Δ0 *met15*Δ0). Protein tagging, promoter replacements, and individual gene deletions were performed by standard PCR-based homologous recombination [78,79]. Primers were designed using Primers-4-Yeast [80] for pFA6 and pYM plasmid sets [78,79]. Plasmid Opi1-GFP, under its own promoter, was generated from genomic DNA derived from a chromosomally C-terminal GFP-tagged version of Opi1. Amplification was performed using primers Opi1Not1F (CTACCAATGCGGCCGCATGAAAACCTCAATGAATCC; 535 bp upstream of ATG) and Opi1 HindIIIR (TACAATAGAAGCTTACGAGGCAAGCTAAACAGATC), and the insert was cloned into *Not*I and *Hin*dIII sites of pRS415. The promoter of the *DGA1* gene was amplified from genomic DNA and cloned into the *Eco*RI and *Bam*HI sites of the multicopy lacZ reporter YEp357 [81] using primers DGA1pr_EcoRI_Fw (TAAGCAGAATTCGATGAGATTATTGCCTTTACTGCGCCATT) and DGA1pr_BamHI_Rv (TGCTTAGGATCCTCTTCTTATATCATTGAATGTTCCTGACATTTATGTGACTGTTCA). All constructs were verified either by sequencing (plasmids) or PCR (mutant strains). Strains were transformed using the standard lithium acetate procedure [82].

### 3.2. Media and Growth Conditions

Cells were grown in synthetic complete (SC) medium [SC drop-out medium containing 2% (*w*/*v*) glucose, 0.67% yeast nitrogen base without amino acids (Difco Laboratories, Detroit, MI, USA) and supplemented with appropriate amino acids (80 mg histidine L^−1^, 400 mg leucine L^−1^, 80 mg tryptophan L^−1^, and 80 mg uracil L^−1^)]. For the nitrogen starvation assay, cells were grown in 2% (*w*/*v*) glucose, 0.17% yeast nitrogen base without amino acids, and ammonium sulfate (Difco Laboratories). Citrate (sodium citrate and citric acid solution; pH 4.3 and 5.2; Sigma-Aldrich, St. Louis, MO, USA) and 3-(N-morpholino)propanesulfonic acid (MOPS; pH 6.6; NZYTech, Lisbon, Portugal) buffers were used to a final concentration of 100 mM in media. The pH of the media was adjusted with either HCl or NaHO. Cultures were routinely grown in an orbital shaker at 140 r.p.m., at 26 °C. Cerulenin was purchased from Cayman Chemical, Ann Arbor, MI, USA, and rapamycin, myo-inositol, choline, and carbonyl cyanide 3-chlorophenylhydrazone (CCCP) were acquired from Sigma-Aldrich, St. Louis, MO, USA. Where indicated, the FA synthesis inhibitor cerulenin was added to cultures to a final concentration of 10 µg/mL. Myo-inositol and choline were used at a final concentration of 1 mM, except for the shifting medium β-galactosidase activity assay (75 µM). Rapamycin was used at a final concentration of 200 ng/mL and CCCP at a final concentration of 10 μM, both dissolved in DMSO.

### 3.3. Targeted Screen for Regulators of LD Dynamics and Flow Cytometry Assays

A collection of pre-defined all non-essential yeast deletion strains was used. Overnight precultures grown in SC medium were diluted to optical density OD_600_ = 0.15 and allowed to grow for 31–32 h to reach the early stationary phase. 0.5 OD cells were harvested by centrifugation and washed twice with phosphate-buffered saline (PBS). LDs in cells from the stationary phase were labeled with BODIPY 493/503 (Invitrogen, Waltham, MA, USA; final concentration of 1 µg/mL) in PBS buffer, in the dark and at room temperature for 10 min. Cells were washed 2 times with PBS and resuspended in 400 µL of PBS. BODIPY493/503 mean fluorescence intensity (MFI) was analyzed using a BD Accuri C6 flow cytometer. BODIPY 493/503 was excited by the 488-nm laser and detected in the FL1 channel. A total of 50,000 events were analyzed per strain. Data analysis was performed using FlowJo v10.

To measure Opi1-GFP levels, WT, *vma1*Δ, and *pma-1007* cells expressing Opi1-GFP were grown in SC medium lacking leucine to early stationary phase, and fluorescence intensity analysis of the GFP signal was performed by flow cytometry (FL1 channel).

For glucose starvation, cells were grown in buffered SC medium (pH 5.2) to exponential phase. The culture was divided in 2 batches, and cells were harvested by centrifugation and washed twice with water. Cells were then resuspended either in fresh SC medium (pH 5.2) or SC medium without glucose (pH 5.2), and aliquots were collected at 0, 30, and 60 min. BODIPY493/503-fluorescence intensity at the indicated times was measured by flow cytometry, as described above.

For the lipolysis assay, overnight precultures grown in SC medium were diluted to OD_600_ = 0.2, and then allowed to grow for 5 h in fresh SC medium containing cerulenin. LD BODIPY493/503-fluorescence at time 0 and after 5 h growth in fresh medium containing cerulenin was measured by flow cytometry, as described above.

### 3.4. Quantification of Neutral Lipids

To quantify TAG and SE levels, cells were grown overnight to saturation in SC with amino acids (US Biologicals, Salem, MA, USA) at 26 °C. The next day, 10 mL cultures were started in the same medium with cells at OD_600_ = 0.15 and 50 Ci [^3^H]acetate (American Radiolabeled Chemicals, St. Louis, MO, USA) and grown for 31 h at 26 °C. The lipids were extracted as described [83], and spotted onto silica gel 60 thin layer chromatography (TLC) plates (Merck, Darmstadt, Germany). The plates were developed with hexane-diethylether-acetic acid (70:30:2) and quantified using a RITA Star Thin Layer Analyzer (Raytest, Straubenhardt, Germany). The amounts of TAG and SE were normalized to the total radioactivity of the sample.

### 3.5. Western Blotting

To monitor TORC1 activity, we evaluated the phosphorylation of ribosomal protein S6 (Rps6) during growth, as described [84]. Briefly, cell pellets were resuspended in lysis buffer (50 mM Tris-HCl pH 7.5, 150 mM NaCl, 15% glycerol, 0.5% Tween-20, phosphatase inhibitor mixture and EDTA-free protease inhibitor cocktail, Roche, Basel, Switzerland) and total protein extracts were obtained by mechanical disruption through vigorous shaking of the cell suspension in the presence of glass beads for 5 min. Short pulses of 1 min were applied, followed by 1 min incubation on ice. Cell debris was removed by centrifugation at 13,000 r.p.m. for 15 min (4 °C), and protein content was determined by the method of Lowry, using bovine serum albumin (Sigma-Aldrich, St. Louis, MO, USA) as a standard. Proteins were then analyzed by SDS-PAGE using 10% polyacrylamide (Sigma-Aldrich, St. Louis, MO, USA) gels and blotted onto a nitrocellulose membrane (GE Healthcare, Little Chalfont, UK). Immunoblotting was performed using the anti-phospho-Ser235/Ser236-S6 Ribosomal Protein (rabbit polyclonal; #2211, Cell Signalling Technology, Danvers, MA, USA) at a final dilution of 1:5000, and anti-Pgk1 (mouse monoclonal; clone 22C5D8; #459250, Invitrogen, Waltham, MA, USA) at a final dilution of 1:15,000. Immunoblot was revealed by chemiluminescence (ECL, Advansta, San Jose, CA, USA).

### 3.6. Measurement of Enzymatic Activities

For the β-galactosidase assay, cells expressing *DGA1*-LacZ were grown to exponential phase in SC medium lacking uracil or overnight precultures were diluted to OD_600_ = 0.15 and allowed to grow for 32 h to reach early stationary phase. For the *INO1*-LacZ gene reporter, cells were grown to an exponential phase in buffered SC medium lacking leucine without inositol (pH 4 and 5). For shifting cells from medium containing inositol to medium lacking inositol, overnight cultures grown in buffered (pH 5) SC medium lacking leucine supplemented with inositol (75 μM) and choline (I^+^C^+^) were diluted to OD_600_ = 0.2 in the same medium. Cells were allowed to grow to mid-exponential phase. Cells were harvested by centrifugation, washed twice with water, and then resuspended in buffered (pH 5) SC medium lacking inositol and supplemented with choline (I^−^C^+^), and incubated for 2 h. β-galactosidase activity was measured at 30 °C using the substrate *o*-nitrophenyl-β-D-galactopyranoside (ONPG; Merck, Kenilworth, NJ, USA), as described previously [85].

### 3.7. Measurement of Cytosolic pH

Cytosolic pH measurements were performed using the same amounts of cells resuspended in the corresponding buffered SC medium (1 OD cells in 500 µL) [86,87,88]. In parallel, to obtain the calibration curve for each experiment, WT cells expressing pHluorin (transformed with pYB1903) were resuspended in 500 μL of a series of calibration buffers [50 mM 2-(N-morpholino)ethanesulfonic acid (MES, Sigma-Aldrich, St. Louis, MO, USA), 50 mM 2-[4-(2-hydroxyethyl)piperazin-1-yl]ethanesulfonic acid (HEPES, Merck, Kenilworth, NJ, USA), 50 mM sodium chloride (NaCl, Acros Organics, Fair Lawn, NJ, USA), 200 mM ammonium acetate (Sigma-Aldrich, St. Louis, MO, USA), 10 mM sodium azide (NaN_3_, Sigma-Aldrich, St. Louis, MO, USA), and 10 mM 2-deoxy-D-glucose (Sigma-Aldrich, St. Louis, MO, USA)] adjusted to pH 5.0, 5.5, 6.0, and 7.0, and incubated at 26 °C for 30 min. To measure cytosolic pH, cells transformed with pYB1903 were grown to an exponential phase in buffered SC medium lacking uracil (pH 4 and 5). 100 μL of each cell suspension (calibration or experimental samples) were transferred to a 96-well flat-bottom microtiter plate (Nunc, Roskilde, Denmark) in duplicate. pHluorin fluorescence emission intensity (*I*) at 510 nm after excitation to 390 nm (*I_390_*) and 475 nm (*I_475_*) was acquired using a microwell plate reader (BioTek^®^ Synergy™ Mx, Winnoski, VT, USA). To obtain the calibration curve, fluorescence intensity signals were background-subtracted using data from the untransformed, pHluorin-free samples. For each intensity recorded for the strains, a blank value corresponding to the culture medium was also subtracted. A calibration curve of the ratio *I_390_* /*I_475_* versus pH was obtained. To estimate cytosolic pH from experimental samples, *I_390_* /*I_475_* ratios were used to obtain pH values according to the calibration curve.

### 3.8. Fluorescence Microscopy

Cells were visualized and imaged live using Zeiss Axio Imager Z1 microscope (Carl Zeiss, Jena, Germany) operated with the Axiovision 4.9 software (Carl Zeiss), a Plan-Apochromat 63x/1.40 Oil DIC, and an Axiocam MR ver3.0 (Carl Zeiss) camera, and processed using Fiji/ImageJ software (version 2.0.0). BODIPY493/503, and GFP signals were detected using a GFP filter with standard settings. Images being directly compared were obtained using identical microscope settings. For quantification of LD number, we performed automated analysis and defined the LD index. For this purpose, stacks of images were processed by deconvolution and maximal projection using Fiji/ImageJ software. LDs were defined as objects/particles and analyzed using the Fiji particle analysis automated procedure (https://imagej.nih.gov/ij/docs/menus/analyze.html, accessed on 9 January 2020). Composite images were generated with the DIC channel to score the total cell number. The total number of objects calculated by the software, divided by the total cell number, defines the LD index. A higher LD index indicates a higher number of LDs per cell for the strain evaluated. For localization of Opi1-GFP and EGFP-RBD-3 reporter, planes were recorded, and the distribution of the GFP signal within the cell was inspected. The vacuole was assigned based on the DIC signal of the corresponding position in the z-axis. Cells were washed twice with PBS, pH 7.4, before visualization. When applicable, all quantifications were performed from at least 2 independent experiments, and more than 100 cells/conditions were scored. Values were recorded in Excel (Microsoft) and analyzed in Prism 8.0 (GraphPad Software). Brightness and contrast were adjusted using Inkscape (The Inkscape Project).

### 3.9. Bioinformatics Analysis

In-silico analysis of transcriptional regulation of the *DGA1* promoter was performed using the Yeast Search for Transcriptional Regulators and Consensus Tracking (YEASTRACT) database [42]. The search was carried out to assess regulatory associations between the TFs and *DGA1* promoter, either document or potential associations (based on TF binding sites). The Bayesian phylogenetic tree was produced using the ADOPS (Automatic Detection of Positively Selected Sites) pipeline [89]. In this pipeline, nucleotide sequences were first translated and aligned using the amino-acid alignment as a guide. The MUSCLE alignment algorithm was used as implemented in T-Coffee [90]. Only codons with a support value above 2 were used for phylogenetic reconstruction. MrBayes 3.1.2 [91] was used as implemented in the ADOPS pipeline. The general time-reversible model (GTR) of sequence evolution was implemented in the analysis, allowing for among-site rate variation and a proportion of invariable sites. Third codon positions were allowed to have a gamma distribution shape parameter different from that of first and second codon positions. Two independent runs of 1,000,000 generations with 4 chains each (1 cold and 3 heated chains) were completed. The average standard deviation of split frequencies was constantly about 0.01, and the potential scale reduction factor for each parameter about 1.00, showing that convergence was succeeded. Trees were tested every 100th generation with a defined burn-in of 25% for the complete analysis (first 2500 samples were discarded). The undiscarded trees were used to compute the Bayesian posterior probability values of each clade of the consensus tree. The Nexus format Bayesian trees produced as output by the ADOPS pipeline were converted to Newick format using the Format Conversion Website (http://phylogeny.lirmm.fr/phylo_cgi/data_converter.cgi, accessed on 5 November 2019). This Newick formatted file was imported to MEGA7 in order to root the consensus phylogenetic tree.

### 3.10. Quantification and Statistical Analysis

Unless otherwise stated, the results were obtained from at least 3 independent experiments. The images were representative of the results obtained. Quantitative results were expressed as mean ± SD. Statistical analysis was performed with unpaired, two-tailed Student’s *t* tests using Prism 8.0 (GraphPad Software). *p* values < 0.05 are considered significant: * *p ≤* 0.05; ** *p ≤* 0.01; *** *p ≤* 0.001; **** *p ≤* 0.0001.

### 3.11. Online Supplemental Material

Figure S1 shows data associated with Figure 1. Data showed no significant changes in growth between strains at the stationary phase. Sit4 regulates Snf1-mediated activation of Acc1 and LD generation. Glucose starvation transiently induces LD generation. Figure S2 shows data associated with Figure 2, Figure 3, and Figure 5. LD number in *vma1*Δ and *pma1-007* mutants was increased. CCCP-treated cells had increased LD generation, irrespective of extracellular pH. The Bayesian phylogenetic tree revealed that *S. cerevisiae* Opi1 sequences were represented as a sister group relative to mouse and human IP6K1 sequences. Figure S3 shows data associated with Figure 5. Loss of V-ATPase activity renders cells inositol auxotrophic, and this phenotype was rescued upon *OPI1* deletion. When *OPI1* expression was restored in the *vma1*Δ*opi1*Δ double mutant, it became inositol auxotroph, indicating that Opi1 contributes to this phenotype. Opi1-mediated transcriptional repression in *vma1*Δ cells was not responsive to alterations in PA levels.

## 4. Conclusions

In summary, our study addresses the interplay among lipid metabolism, nutrient sensing signaling pathways, and Opi1-mediated transcriptional activity (Figure 6C). It has significant implications for our understanding of how master regulators such as Ras-PKA, TORC1, V-ATPase, and Pma1 fit within the signaling processes that control transcription of lipid metabolic genes involved in membrane biogenesis and stress response. Importantly, the functional crosstalk between lysosomes and LDs has been unraveled in the past few years, particularly at the level of lipophagy and lysosomal acid lipolysis, which goes beyond ordinary functions played by these organelles in cellular metabolism [92]. This ranges from antigen presentation by adipocytes [93] in the setting of obesity-related inflammation [94], to liver disease [95] and lysosomal storage diseases [96]. Overall, this work provides additional insights that link changes in activation of major signaling effectors and V-ATPase to lipid dyshomeostasis and lysosomal dysfunction associated with intracellular pH deregulation, a major metabolic feature observed in disease pathology across the age spectrum [15,56,97,98].

## Data Availability

Not applicable.

## Supplementary Materials

The following are available online at https://www.mdpi.com/article/10.3390/ijms22169017/s1, Figure S1. Convergence of TORC1-Sit4 and Snf1-glucose signaling onto LD metabolism, Figure S2. Aberrant LD generation occurs in response to loss of intracellular pHc homeostasis, Figure S3. V-ATPase activity regulates inositol metabolism and Opi1-mediated control of *INO1* transcription, Figure S4. The raw data of Western blot assays, Table S1A. List of potential transcription factors (TF) that have a binding sequence in the *DGA1* promoter regions (first 1000 bp upstream of the START codon), according to YEASTRACT+ [42], Table S1B. List of documented regulations, according to YEASTRACT+ [42], and filtered by DNA binding plus expression evidence and TF acting as activator or inhibitor, Table S2. *S.cerevisiae* strains and plasmids used in this study.
