# Supplementary material for: Target of Rapamycin Complex 1 (TORC1), Protein Kinase A (PKA) and Cytosolic pH Regulate a Transcriptional Circuit for Lipid Droplet Formation
Source: Int J Mol Sci. 2021 Aug 20;22(16):9017. doi: 10.3390/ijms22169017 (PMC8396576; doi:10.3390/ijms22169017)
Supplement: Supplementary file 1 [file ijms-22-09017-s001.zip › Figure S1_IJMS.pdf]

Figure S1

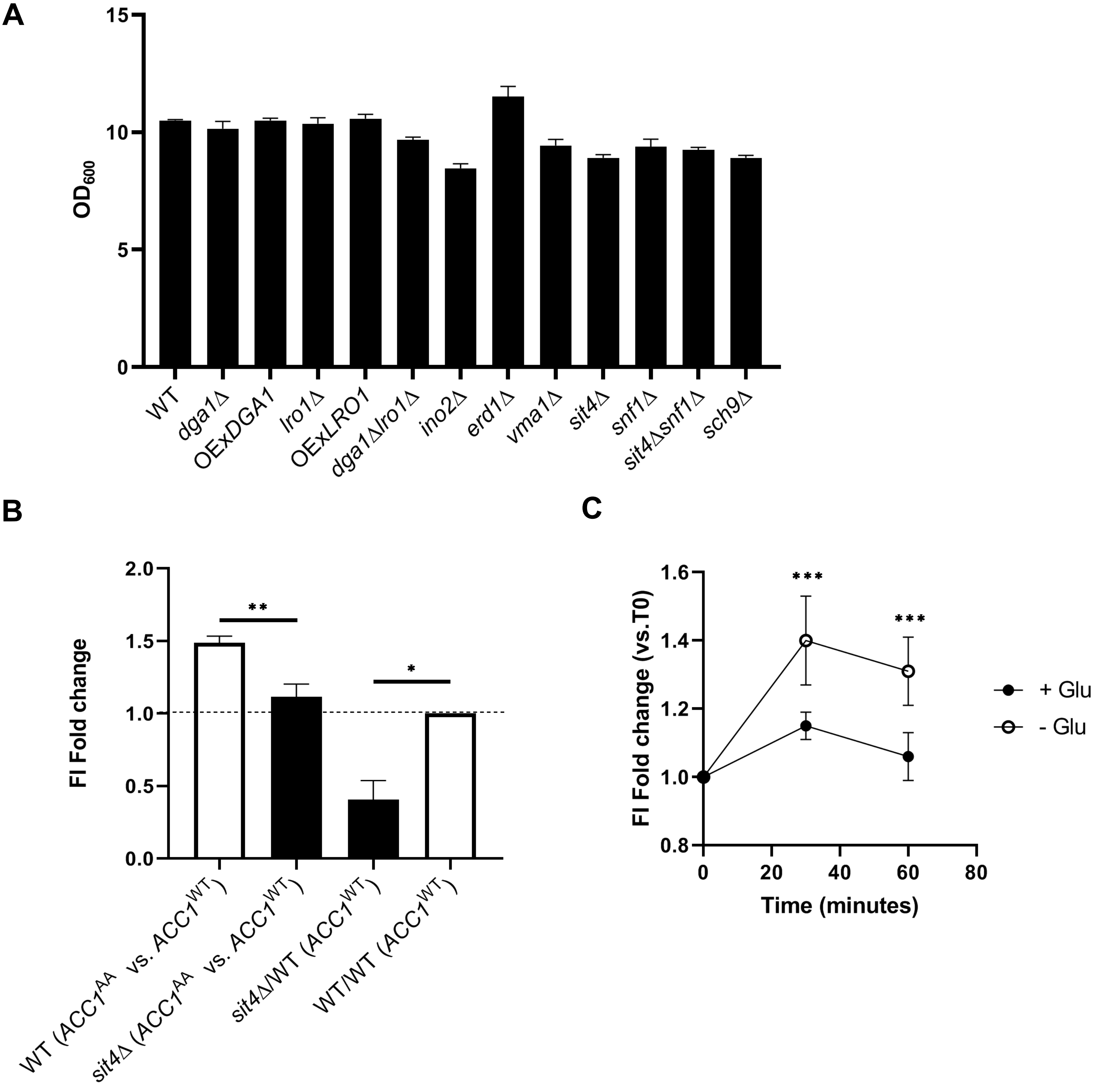

Figure S1. Convergence of TORC1-Sit4 and Snf1-glucose signaling onto LD metabolism. (A) No significant changes in growth between WT and indicated genotypes at stationary phase were detected. **(B)** Expression of mutant *ACC1*, with two site mutations at serines 659 and 1157 ($ACC1^{Ser659Ala,Ser1157Ala}$, $ACC1^{AA}$), promotes LD induction in WT cells, thus recapitulating the LD-inducing phenotype observed in snf1Δ cells (Figure 1B). Unlike WT, *sit4*Δ cells, where Snf1-mediated phosphorylation and inhibition of Acc1 activity is expected, the expression of this allele has no effect. FI ratios for each condition are shown between strains. **(C)** Glucose starvation transiently promotes LD generation. Cells grown to exponential phase were collected, washed with water, and then resuspended either in fresh SC medium (pH 5.2) or SC medium without glucose (pH 5.2), and aliquots were collected at 0, 30 and 60 min. BODIPY493/503-fluorescence at the indicated times was measured by flow cytometry as described in Figure 1B. For each condition, shown are FI fold changes vs. time 0. Results are mean±SD of at least three independent experiments. *$P\leq0.05$; **$P\leq0.01$; ***$P\leq0.001$.
