# Supplementary material for: Target of Rapamycin Complex 1 (TORC1), Protein Kinase A (PKA) and Cytosolic pH Regulate a Transcriptional Circuit for Lipid Droplet Formation
Source: Int J Mol Sci. 2021 Aug 20;22(16):9017. doi: 10.3390/ijms22169017 (PMC8396576; doi:10.3390/ijms22169017)
Supplement: Supplementary file 1 [file ijms-22-09017-s001.zip › Figure S2_IJMS.pdf]

Figure S2

A

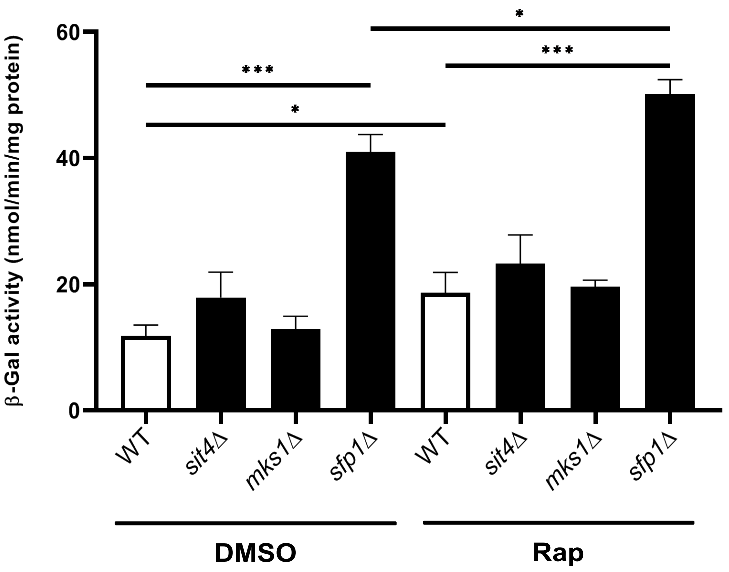

B

Strain	LD index (objects per cell)
**WT**	2,5
vma1Δ	3,6
pma1-007	3,2

C

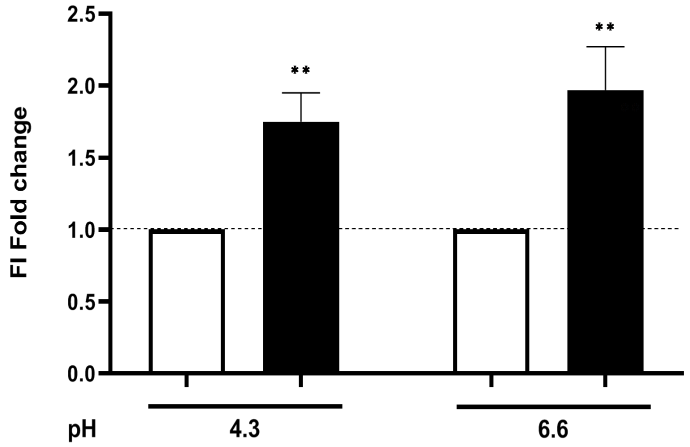

D

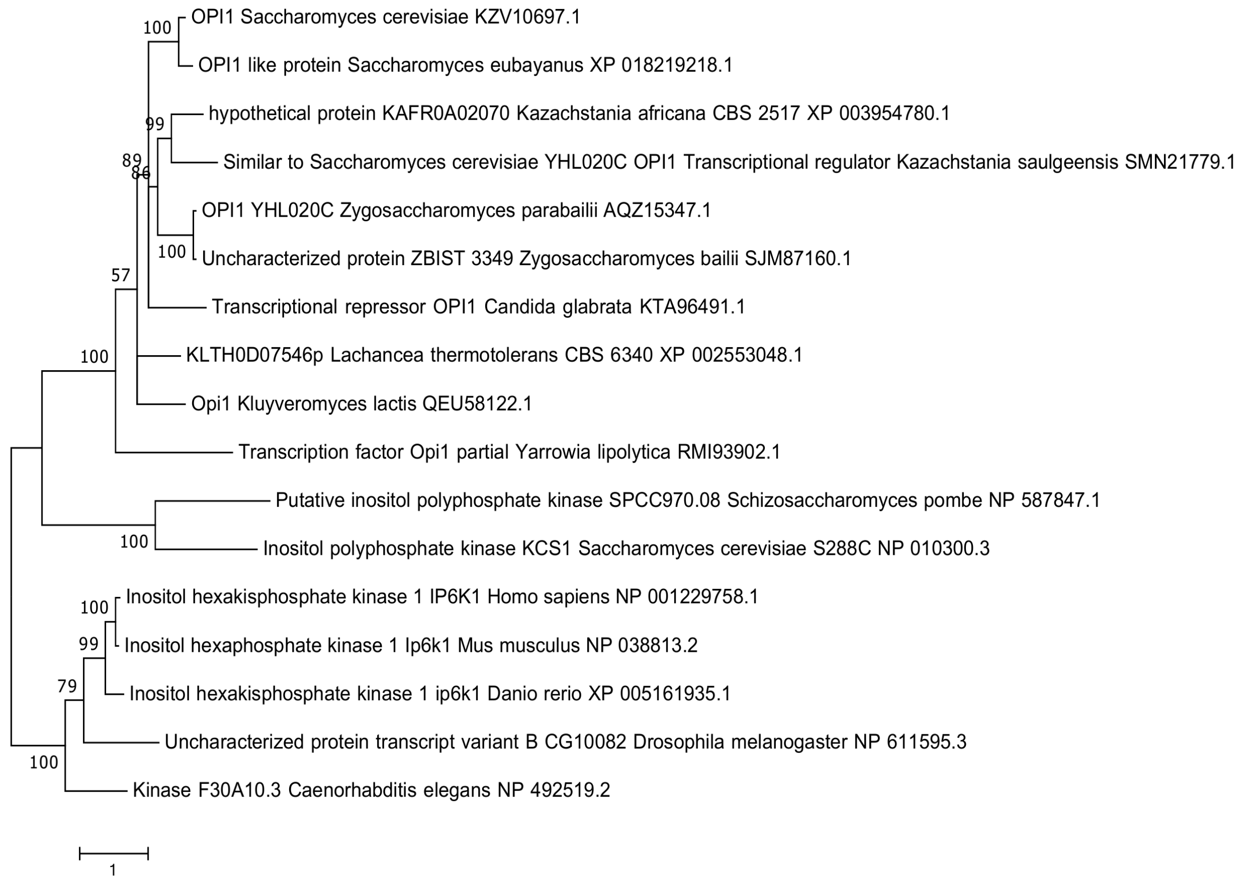

Figure S2. Aberrant LD generation occurs in response to loss of intracellular pH homeostasis. (A) Cells expressing YEp357-*DGA1*-LacZ were grown in SC medium lacking uracil to exponential phase and then treated with rapamycin (Rap, 200 ng/mL) or vehicle (DMSO) for 2 hours. Protein extracts were prepared, and specific β-galactosidase (β-Gal) activities, with o-nitrophenyl-β-D-galactopyranoside (ONPG) as a substrate, were determined by measuring the amount of o-nitrophenol released by the galactosidase-catalyzed hydrolysis process. **(B)** *vma1*Δ and *pma1-007* cells display higher number of LDs, as defined by LD index (described in Materials and Methods). Cells were grown as described in Figure 1B. **(C)** Treatment with proton ionophore carbonyl cyanide 3-chlorophenylhydrazone (CCCP), which disrupts plasma membrane proton gradient, leads to increased fat content (black bars), irrespective of extracellular pH, thus phenocopying the *pma1-007* mutation. BODIPY493/503-associated fluorescence intensity (FI), which is a measure of LD content, was monitored by flow cytometry in the FL1 channel. For each condition, shown are FI fold changes vs. WT in the absence of CCCP. **(D)** The Bayesian phylogenetic tree revealed that *S. cerevisiae* Opi1 sequences are represented as a sister group relative to mammalian IP6K1 sequences. Results are mean±SD of at least three independent experiments. *$P \leq 0.05$; **$P \leq 0.01$; ***$P \leq 0.001$.
