# Supplementary material for: Target of Rapamycin Complex 1 (TORC1), Protein Kinase A (PKA) and Cytosolic pH Regulate a Transcriptional Circuit for Lipid Droplet Formation
Source: Int J Mol Sci. 2021 Aug 20;22(16):9017. doi: 10.3390/ijms22169017 (PMC8396576; doi:10.3390/ijms22169017)
Supplement: Supplementary file 1 [file ijms-22-09017-s001.zip › Figure S3_IJMS.pdf]

Figure S3

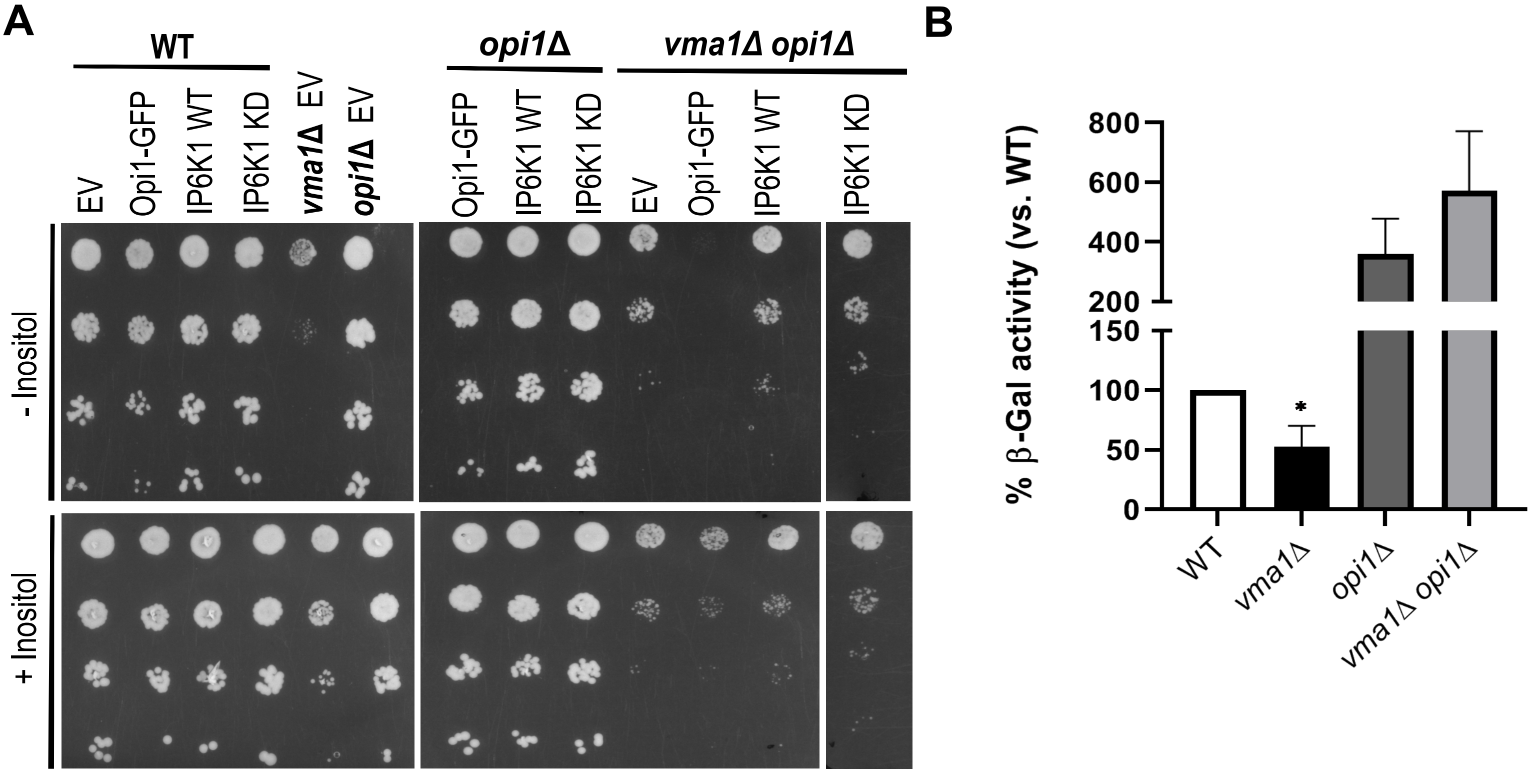

Figure S3. V-ATPase activity regulates inositol metabolism and Opi1-mediated control of *INO1* transcription. (A) Loss of V-ATPase activity renders cells inositol auxotrophic, and this phenotype was rescued upon *OPI1* deletion. Re-expression of *OPI1* in the *vma1*Δ*opi1*Δ double mutant restored inositol auxotrophy, confirming that Opi1 contributes to this phenotype. Cells of indicated genotypes were grown in SC medium lacking leucine and inositol to the exponential phase and diluted to OD600=0.1. Tenfold dilution series were spotted onto SC plates lacking leucine and inositol, or supplemented with inositol when indicated. Plates were incubated at 26°C. **(B)** Opi1-mediated transcriptional repression in *vma1*Δ cells is not responsive to alterations in PA levels induced by a shift to inositol-free medium. Results are shown as a percentage of WT levels. Results are mean±SD of at least three independent experiments. *$P \leq 0.05$.
