# Supplementary material for: Target of Rapamycin Complex 1 (TORC1), Protein Kinase A (PKA) and Cytosolic pH Regulate a Transcriptional Circuit for Lipid Droplet Formation
Source: Int J Mol Sci. 2021 Aug 20;22(16):9017. doi: 10.3390/ijms22169017 (PMC8396576; doi:10.3390/ijms22169017)
Supplement: Supplementary file 1 [file ijms-22-09017-s001.zip › Figure S4_IJMS.pdf]

Figure S4

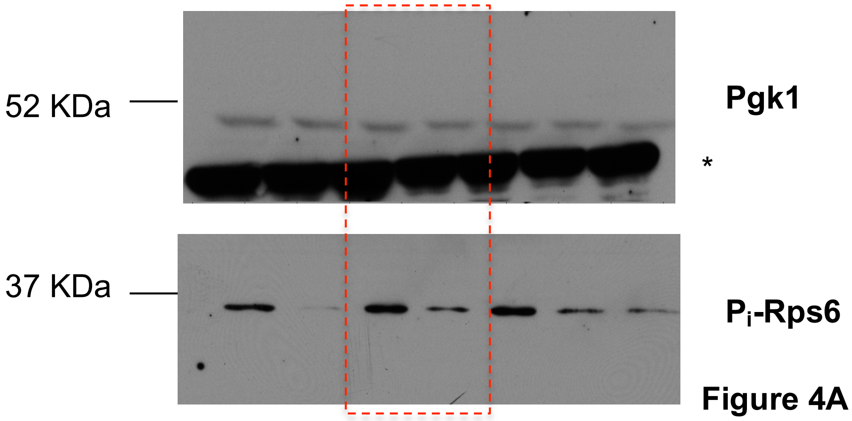

Figure 4A

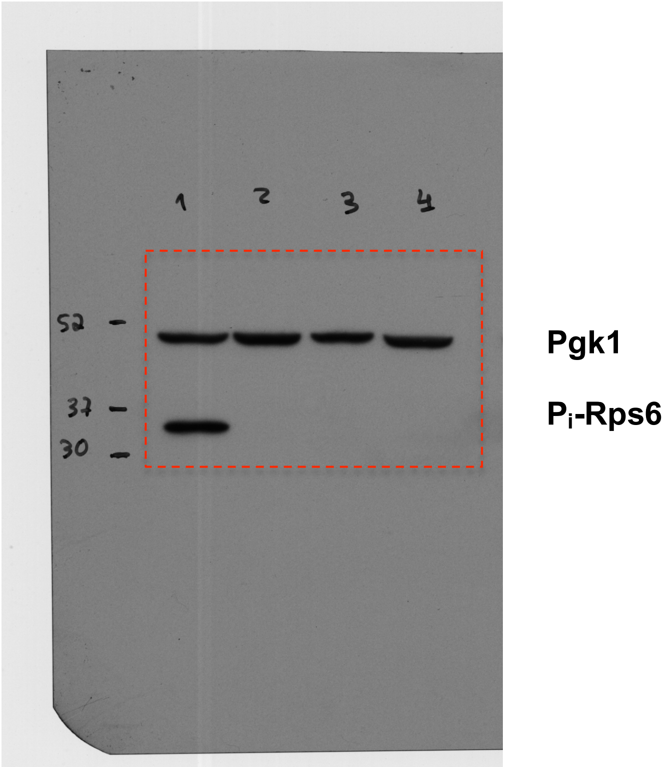

Figure 2B

Supplementary Figure S4. The raw data of Western blot assays. The figures display all blots used in this study and in dashed red the selected areas shown in the main text. * unspecific band.
